# Unlocking the regulatory code of RNA: launching the Human RNome Project

**DOI:** 10.1186/s13059-025-03824-y

**Published:** 2025-10-24

**Authors:** The International Human RNome Project Consortium, Mark D. Adams, Etienne Boileau, Janusz M. Bujnicki, Vivian G. Cheung, Silvestro G. Conticello, Peter Dedon, Christoph Dieterich, Angela Gallo, Jonathan Göke, Mark Helm, Michael F. Jantsch, Stefanie Kaiser, Charles Lee, Virginie Marchand, Ali Mortazavi, Yuri Motorin, Schraga Schwartz, Blanton S. Tolbert, James Williamson

**Affiliations:** 1 Brown University, Providence, RI, USA; 2 Goethe-University Frankfurt, Frankfurt, Germany

## Abstract

The human RNome, the complete set of RNA molecules in human cells, arises through complex processing and includes diverse molecular species. While research traditionally focuses on four canonical nucleotide residues, the RNome, encompassing over 180 distinct modifications across organisms, with at least 50 in humans, is increasingly recognized. These modifications play critical roles in regulating RNA structure, stability, and function, yet the rules linking their precise locations to biological outcomes remain poorly defined. The Human RNome Project aims to map all RNA modifications, build essential resources, and harness new technologies to transform RNA biology, therapeutic development, agriculture, and even data storage.

## Introduction

**Fig. 1:**
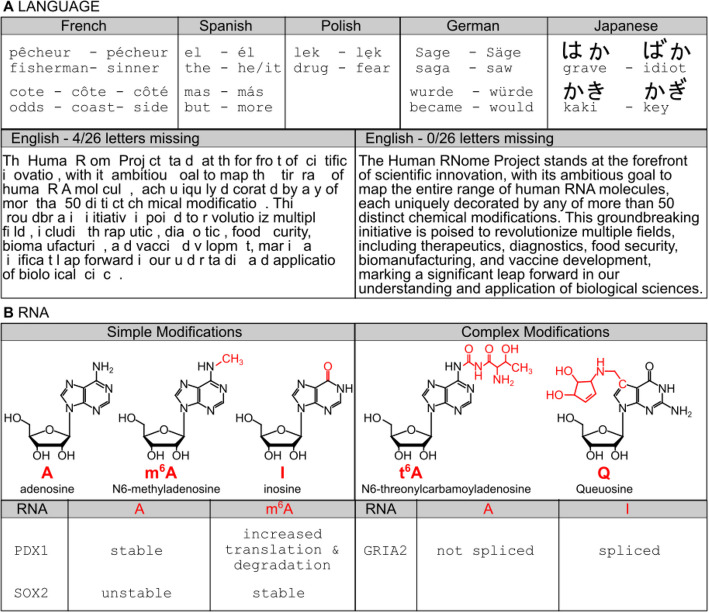
Analogy of language and RNA modifications.** A** The human RNome is difficult to read and understand if diacritic marks (top) or letters (bottom) are missing. The top panel shows words in French, Spanish, Polish, German, and Japanese that change their meaning due to diacritic marks (English translation below). On the bottom, 4 letters of the alphabet were removed to showcase the drastic effects in the English language (namely n, e, s, and k). **B** Chemical structures of simple RNA modifications of adenosine that equal diacritic marks in language and more complex modifications that can be viewed as additional letters of the RNA language. Bottom: Example transcripts that change in, e.g., translation, stability, and splicing in dependence of RNA modifications (PDX1 [3, 4], SOX2 [5–7], and GRIA2 [8–10])

RNA is a multifunctional polymer, essential for defining cell identity and structure, regulating biological processes, and responding to environmental stimuli. Although transcribed from DNA, RNA is extensively processed and modified co- and post-transcriptionally. With wide variation among organisms, these modifications include splicing, 5′-capping and 3′-polyadenylation [1, 2], and diverse enzymatic modifications of ribonucleotide components. These processing steps shape RNA’s structure and regulate its interactions with proteins and other nucleic acids, underscoring RNA’s critical role in cellular function and adaptability (Fig. 1).

Our understanding of the complete set of RNA molecules in a cell, the RNome, becomes greatly more complicated when considering that humans are composed of 200 different cell types organized as 79 organs [11]. RNA expression patterns dictate the varying functions of these cells. While existing RNA sequencing (RNA-seq, more appropriately cDNA sequencing) technologies are adequate to identify and quantify the full set of RNA transcripts in a cell, tissue, or organ, the presence of over 180 enzymatic modifications of RNA across all organisms—the epitranscriptome—greatly complicates this picture [12, 13]. The ~ 50 RNA modifications in humans [12–14] play critical roles in RNA biology and disease, yet the rules linking the location of RNA modifications to RNA structure and function are poorly defined. This becomes even more complicated as the epitranscriptome roster continues to grow, such as the new wobble modification ava^2^C [15]. A biogeographical map of RNA modifications is thus essential for fundamental understanding of RNA biology and for enabling future RNA-centered applications. The major impediment to a comprehensive study of the RNome is that conventional next-generation RNA sequencing methods do not actually sequence RNA, but rather convert RNA into cDNA, a process that removes information about modifications [16]. Third-generation or direct RNA-seq, such as that offered by Oxford Nanopore Technologies, partially addresses this limitation by localizing RNA modifications in long sequence reads but the technology suffers from high error rates, limited single-base resolution, a lack of chemical specificity, weak quantification, and the ability to detect only a small subset of modifications. On the other hand, quantitative and chemically specific RNA-seq technologies such as liquid chromatography-coupled mass spectrometry (LC–MS) are restricted to short RNA fragments and incapable of full-length sequencing with complete chemical annotation.

To address these technological barriers, the International Human RNome Project Consortium was established in 2024 with the goal of defining the research infrastructure, standards, and protocols for sequencing all RNAs and mapping their enzymatic modifications. The Consortium identified four foundational steps to guide this effort: (i) identifying key RNA species and cell types for sequencing, (ii) sourcing molecular resources and standards, (iii) establishing advanced analytical technologies, and (iv) developing guidelines for data analysis, formatting, and storage. These preliminary steps aim to establish a robust framework for mapping the RNome, a task that surpasses the complexity of genome sequencing due to the dynamic nature of the RNA and its extensive regulatory modifications.

### Defining the RNome

The RNome refers to the complete repertoire of RNA transcripts that dynamically adjust to regulate cellular needs. While all cells in an organism share the same genome, their RNomes differ significantly, with 200–300,000 different transcripts shaping cell identity and fulfilling specific cellular and organismal requirements. Unlike the static DNA sequences, RNA molecules are inherently dynamic, changing in response to cellular states.

Efforts like the Human Genome Project [1, 2] and 1000 Genomes Project [17, 18] successfully sequenced the genomes of individual humans, with annotation of these genes pursued by groups such as Gencode [19], Ensembl [20], RefSeq [21], and CHESS [22]. However, as described by a recent National Academies of Sciences, Engineering and Medicine consensus report [23], there is no comparable initiative for the human RNome due to its complexity and variability. The Genotype-Tissue Expression (GTEx) project [24], the Encyclopedia of DNA Elements (ENCODE [25, 26]), and the Human Cell Atlas Project [27] are mapping the variability among human cell types and tissues, but are limited to the transcriptional layer. The Human RNome Project seeks to address this gap by providing baseline data that includes the actual sequences including the chemical modifications. Unlike conventional RNA-seq, which converts RNA into cDNA and loses native modifications, this project will sequence RNA while preserving the full-length transcripts and all the associated modifications.

Given the RNome’s dynamic nature, the development of cost-effective, accurate, and accessible technologies for sequencing, analyzing, and sharing RNA data is critical. A fitting analogy of the function and importance of RNA modifications is found in many human languages (Fig. 1). Small RNA modifications, e.g., methyl marks, can be viewed as diacritic marks in languages such as French, Spanish, Polish, German, or Japanese. Here, the addition of the “diacritic = modification” mark can change the whole meaning of a word which is also true for RNA. More complex RNA modifications can be viewed as an addition to the well-known four letters A, U, G, and C previously used to spell words in the RNome. Adding ~ 50 (just human, 180 for all organisms studied so far) more letters and/or diacritic marks in the form of enzymatic modifications dramatically increases the complexity of the RNA dictionary. However, we do not yet have a Rosetta Stone for the human RNome and cannot find one as long as we do not understand and know the human epitranscriptome. Technologies to sequence the RNA and map RNA modifications will empower researchers to decode the RNome of diverse cells and contexts, building comprehensive datasets that unveil the RNA epitranscriptomic regulatory code. This knowledge will not only transform our understanding of RNA biology but also catalyze breakthroughs in precision medicine, sustainable agriculture, and innovative technologies such as RNA-based data storage. To achieve this, we have identified the following critical goals and milestones.

## Identify key RNA species and cell types for sequencing

The Human RNome Project aims to ensure consistent and reproducible outcomes in RNA sequencing and modification studies by utilizing standardized cell lines maintained under uniform culture conditions. This standardized approach will facilitate meaningful comparisons across technologies and laboratories. The selected cell lines will be widely accessible, easy to maintain in culture, and highly proliferative, ensuring an adequate supply of RNA for sequencing and characterization experiments. Importantly, these cell lines will exhibit genetic stability, characterized by a well-defined genome with minimal mutations and chromosomal aberrations, to guarantee the reliability and robustness of the generated data.

To maintain genomic integrity, cell lines will be sourced from certified distributors at regular intervals and used at low passage numbers (< 8). Genetic integrity will be independently verified through DNA and cDNA sequencing, with results reported alongside direct RNA sequence data. This ensures that any genomic drift is identified and accounted for in downstream analyses.

Table 1 lists cell lines that meet these criteria. These lines have been extensively characterized by large-scale studies such as the ENCODE Project [25, 26] and the 1000 Genomes Project [17, 18]. For instance, GM12878, a cultured B-cell line from a female donor with ancestry from Northern and Western Europe, has been sequenced as part of the 1000 Genomes Project and characterized by ENCODE. IMR-90 lung fibroblasts, BJ foreskin fibroblasts, and H9 human embryonic stem cells are similarly well-characterized and available through trusted sources like Coriell, ATCC, and WiCell, which will also enforce standardized protocols for culturing and handling. Given the sensitivity of RNA to environmental factors, these standardizations are critical for ensuring data comparability. Repositories will also require users to follow consistent protocols for culturing and RNA extraction, as variations in these processes could influence RNA sequence and modification profiles.

**Table 1:** Cell lines for initial steps of the Human RNome Project

Cell line	Cell type	Consortia that have studied the cells	Availability
GM12878	B-cells	ENCODE and 1000 Genomes	Coriell Cell Repositories
IMR-90	Lung fibroblast	ENCODE	ATCC
BJ	Foreskin fibroblast	ENCODE	ATCC
H9	Stem cells	ENCODE	WiCell

RNA extraction and quality control: RNA will be extracted using a guanidinium thiocyanate-based method to ensure high purity and integrity. RNA quality will be assessed by absorbance ratio (260/280 and 260/230 nm) and capillary electrophoresis (e.g., Agilent TapeStation), requiring a minimum RNA Integrity Number (RIN) of 9 for RNA extracted from cell lines (as the project advances, and RNA samples are extracted from tissues, a lower RIN threshold such as 8 may be necessary). Aliquots of RNA will be archived for validation and further analyses.

Initial RNA targets for sequencing: The pilot phase of the Human RNome Project will focus on sequencing transfer RNA (tRNA), ribosomal RNA (rRNA), and mRNA, with a focus on selected protein-coding transcripts. These RNA classes are ideal initial targets due to their ubiquity, existing knowledge of their modification profiles, and robust expression across cell types.

### tRNA and rRNA

tRNA (~ 250 expressed isodecoders) and rRNA (5S, 5.8S, 18S, 28S) are universally expressed and highly conserved, with well-studied modification types and locations [12–14]. Table 2 lists examples of modifications typically found in human mRNA, tRNA, and rRNAs. Both total tRNA and rRNAs can be purified from total RNA using electrophoresis or size-exclusion chromatography [28], while affinity-based methods such as chaplet chromatography [29] or reciprocal circulating chromatography [30] can be used to enrich for specific tRNA sequences. One drawback of all RNA purification methods is co-purification of non-target RNAs due to similar size or hybridization to target RNAs. Mass spectrometric analysis of modified ribonucleosides in purified RNA must always be viewed with suspicion for modifications found in multiple forms of RNA (e.g., m^6^A, m^5^C).

**Table 2:** Known modifications in rRNAs and tRNAs as internal validations

RNA type	Known modifications					
rRNAs	Nm	m^6^A	m^7^G	m^3^U	m^5^C	Ψ
tRNAs	Cm, Gm, Um	D	m^7^G	m^1^A/m^1^G	m^5^C	Ψ

### Coding genes and their mRNAs

Selected protein-coding genes include *ACTB*, *CDKN2A*, *ISG15*, and *SOD1*. These genes were chosen based on their known association with diseases, moderate to high expression levels, relatively short transcript lengths (~ 1 kb), and known modifications. For example, SOD1 is associated with amyotrophic lateral sclerosis [31], while ACTB is widely expressed and associated with dystonia (Table 3).

**Table 3:** Protein-coding genes selected as representative RNAs to begin direct RNA sequencing

Gene	Transcript length (bp)	Express in cell lines	m^6^A	Ψ (*)	I	Disease relevance
ACTB	1812	All	Y	Y	Y	Dystonia
CDKN2A	~ 1000	All	Y	nd	N	Cancer
ISG15	867	All	Y	Y	N	Inflammation
SOD1	895	B-cell, HepG2	Y	Y	Y	ALS

^*^Modification information based on information in Sci-ModoM [32]

Coding RNA enrichment methods: To detect low-abundance modifications, enriched RNA samples are critical. Initial poly-A RNA enrichment can be achieved using oligo-dT kits from various vendors [33]. For specific RNAs, biotinylated antisense oligonucleotides allow ~ fivefold enrichment [34], while microbead-based antisense oligos are claimed to achieve a 100,000-fold enrichment [35]. DNA nanoswitches offer another option, with ~ 75% recovery and purities exceeding 99.8% for RNA ranging from 22 to 400 nts [36].

### Future goals

#### Short-term

1. Standardized RNA extraction using guanidinium thiocyanate.
2. Enrichment of test RNAs using antisense-based methods.
3. Mass spectrometry-based direct RNA-seq for short-read identification of modifications and nanopore sequencing for long-read sequencing and modification mapping.

#### Medium-term

1. Sequence transcriptomes from cell sorting-enriched samples of defined cell types.
2. Compare data with existing programs (e.g., GTEx).
3. Expand sequencing to include different cell types and tissues from individuals of all ages and ethnicities.

#### Long-term

1. Sequence RNAs from specific subcellular regions (e.g., nucleus, cytoplasm, mitochondria).
2. Integrate single-cell transcriptomic and subcellular data.

### Source molecular resources and standards

The Human RNome Project relies on robust molecular resources and chemical standards to develop and validate sequencing and mass spectrometry (MS) technologies. These resources encompass synthetic and native RNA standards, as well as their building blocks, such as ribonucleosides, ribonucleotide triphosphates (NTPs), and oligoribonucleotides. High-quality standards are essential for ensuring accurate analysis of RNA modifications, their chemistry, and their precise locations within RNA molecules.

### Chemical standards

**Fig. 2:**
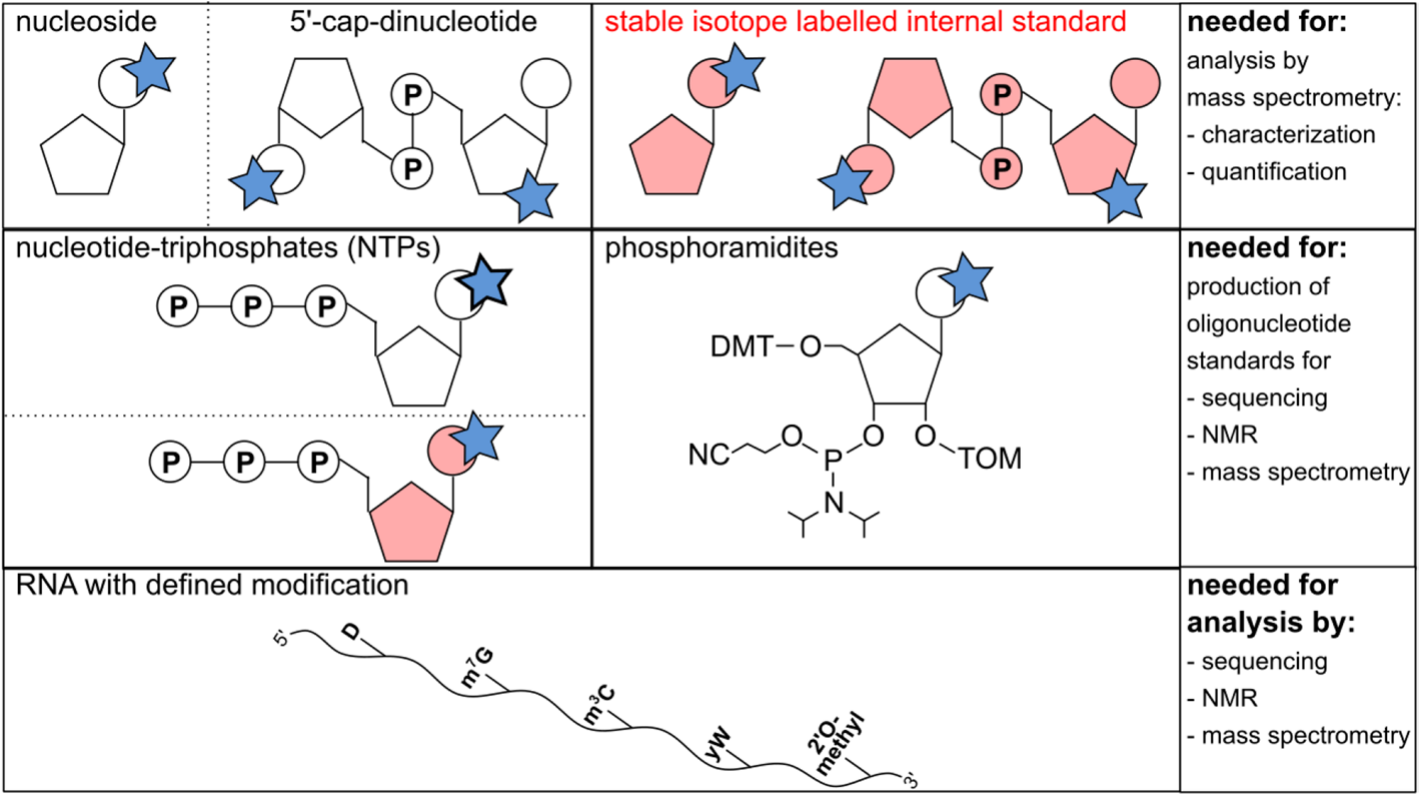
Overview of types of chemical standards needed for the Human RNome Project

Chemical standards are indispensable for training and validating analytical methods before analyzing native RNA samples. They ensure reproducibility, correct identification of RNA modifications, and calibration of detection systems. Standards are summarized in Fig. 2 and include the following.

### Ribonucleosides and dinucleotide caps

Chemical standards for individual ribonucleosides are essential for characterizing RNA modifications and quantifying their abundance. Approximately 90 ribonucleoside standards are commercially available, with additional variants synthesized by academic laboratories. Comprehensive lists of vendors are provided on the RNome website [33], while PubChem offers detailed vendor information and links to chemical resources. Prices for these standards range from $20 to $1500 per milligram, with custom synthesis for rare modifications costing between $10,000 and $20,000. For qualitative analysis, 1 mg of a standard is typically sufficient. For quantitative analysis, we recommend assessing the purity of the standard by quantitative NMR prior to preparing calibration solutions for, as an example, LC–MS analysis. Despite the availability of over 90 modified ribonucleosides, many human-specific RNA modifications remain inaccessible as commercial standards. Furthermore, the chemical stability (shelf life) of ribonucleosides is not well-documented. For example, m^1^A undergoes Dimroth rearrangement to m^6^A during RNA processing and storage in aqueous solution [37, 38] highlighting the need for further research into ribonucleoside stability.

### Ribonucleotide triphosphates

Ribonucleotide triphosphates (NTPs) are essential for in vitro transcription to synthesize RNA molecules longer than 20 nucleotides with defined modification profiles. Canonical NTPs are widely available from commercial sources, including isotopically labeled variants, while modified NTPs for specific ribonucleosides can also be obtained. However, these modified NTPs require rigorous verification of their chemical identity and purity, typically through techniques such as thin-layer chromatography (TLC) or LC–MS [39, 40]. In vitro transcription allows random, but not site-specific incorporation of modified NTPs [41].

### Synthetic oligonucleotides and phosphoramidites

Site-specifically labeled RNA oligonucleotides, ranging from 5 to > 60 nucleotides, are essential for training nanopore base callers and validating LC–MS methods. Solid-phase chemical synthesis is commonly used to produce labeled oligonucleotides and vendors typically provide mass spectra to confirm the overall product length, failure sequences, and impurities. However, comprehensive validation, such as mass spectrometric sequence verification and ribonucleoside LC–MS for modification identification, is rarely included but essential for robust validation. To ensure accuracy, researchers must advocate for detailed validation data, including MS sequence validation and ribonucleoside-specific quantification, alongside the standard mass spectra provided by vendors. Despite these advancements, the site-specific incorporation of modifications into long RNA sequences (> 60 nucleotides) remains a significant challenge [42]. Current approaches, which involve combining chemical synthesis, transcription, and ligation, are labor-intensive, low yielding, and not easily scalable. New approaches to long RNA synthesis are needed to facilitate the generation of site-specifically modified RNAs that mimic biological molecules.

### Future goals

#### Short-term

1. Stability data for modified ribonucleosides is scarce, highlighting the need for systematic studies on shelf life.

#### Medium-term

1. Consistent preparation, validation, and distribution protocols are essential to ensure data comparability over time. Quality control samples must be maintained and shipped with detailed documentation.
2. Researchers should demand comprehensive validation data (e.g., MS/MS, sequence confirmation) from vendors to avoid errors in downstream analyses.

#### Long-term

1. Sequencing and MS methods must be regularly validated using both synthetic and native standards
2. High-quality library of modifications with comprehensive validation data and shelf-lives. Many RNA modifications lack synthetic standards, necessitating collaboration with organic chemists for their production.

#### Develop advanced sequencing technologies

Sequencing technologies will be pivotal to the Human RNome Project, much like they were for the Human Genome Project. To evaluate the potential impact on the project, it is essential to analyze the current state and project developments over the next 5 to 10 years. The consortium has hence identified and discussed lead questions that concern the type of currently available sequencing technologies, the necessary developments in the near future, and critical quality controls.

### Current state of sequencing technologies

Current methods to map modifications can be classified into direct, such as mass spectrometry or direct RNA sequencing, and indirect, which usually relies on sequencing by synthesis, wherein RNA is converted to cDNA via reverse transcriptase [16]. Both indirect and direct RNA sequencing methods require additional steps to assign modifications. This section is meant as a brief summary and not a comprehensive review of all current variations and developments (for a comprehensive review please refer to the Report by the National Academies of Sciences, Engineering and Medicine [23]).

### cDNA-based sequencing

Sequencing of cDNA, acquired through reverse transcription of RNA and analyzed through Illumina (and sometimes PacBio or Nanopore), is currently the most widely used form for indirect RNA sequencing. However, it cannot directly detect non-canonical ribonucleotides. Workarounds to map modifications rely on changing the RNA or cDNA product on a molecular level (“molecular input”) and include reverse transcriptase-based error profiling, chemical or enzymatic derivatization, and modification-specific immunoprecipitation [43, 44]. Molecular input methods utilize computational algorithms that infer RNA modifications from misincorporations, gaps, or reverse transcription arrests or reverse transcription incorporation of structurally similar bases. While powerful, no single molecular input method can comprehensively identify all modifications, necessitating the use of multiple techniques on the same RNA sample.

### Direct RNA sequencing

**Fig. 3:**
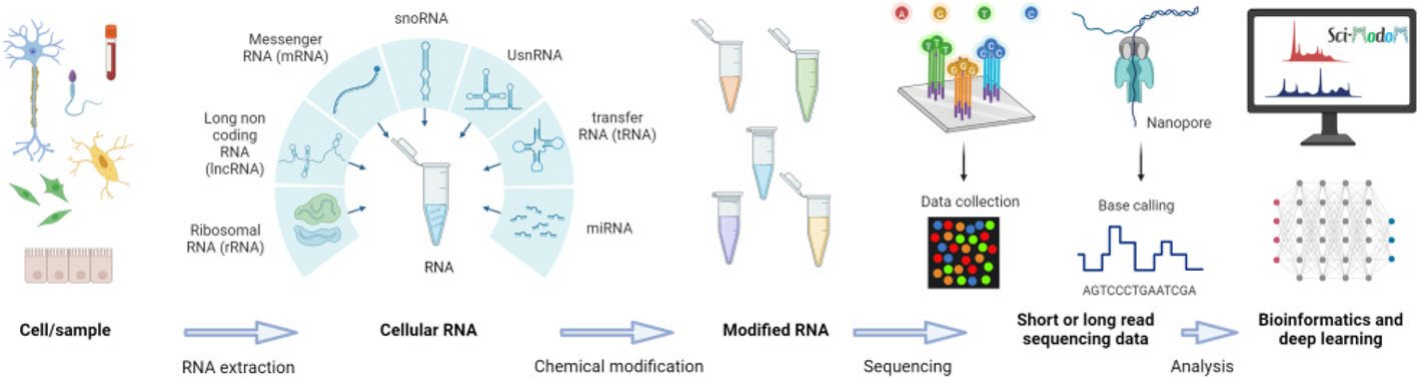
Overview of the sequencing workflow that will allow end-to-end sequencing of RNA including its modifications

Oxford Nanopore Technologies is the only widely available platform currently providing protocols for direct, long-read sequencing of RNA molecules, eliminating the need for cDNA conversion and preserving endogenous or synthetic exogenous RNA modifications (Fig. 3) [45–48]. Advances in machine learning models have led to more accurate basecalling and lower error rates for sequencing full-length native RNA transcripts [47]. By analyzing unique changes in electrical currents from the direct RNA sequencing process, RNA modifications can be tentatively identified [48]. Identification of modified nucleotide residues can be achieved by comparison against unmodified control samples [49], with base-calling algorithms or supervised models that have been trained on data with known modifications [47, 48, 50]. The training of such models can be achieved using data from cDNA-based approaches, data from modification-free control samples [49], in vitro transcription-generated data, or data from synthetic RNAs. However, the generation and availability of such data and the lack of RNA modification standards still limit the number of modifications that can currently be confidently detected and identified. Furthermore, not all reads from direct RNA-seq correspond to full-length RNAs and challenges remain to detect RNA modifications that occur at the 5′ ends of RNA molecules. To overcome these barriers, researchers are actively developing “molecular input” approaches—such as introducing chemical or enzymatic treatments which change the RNA molecule—to amplify or clarify the signals associated with RNA modifications [51].

### Mass spectrometry (MS)

Mass spectrometry (MS)-based RNA sequencing is an essential complement to these efforts as a means to chemically identify and accurately quantify specific modifications [52–55]. Unlike the chemically nonspecific interpretation of electrical signals in nanopore sequencing, MS-based sequencing involves high mass accuracy (i.e., exact molecular weight) determinations of modification fragments that allow structural identification of the modification, its location in the RNA sequence at single-nucleotide resolution, and its abundance in the population of RNA sequences. While MS sequencing requires larger quantities of RNA than NGS or nanopore sequencing, advances in sensitivity have moved the application from more abundant non-coding RNAs to mRNAs [55–58]. The major limitation of MS-based RNA-seq is the short fragment size needed for accurate MS analysis, typically 10–60 nt in length depending upon the mass resolution of the instrument [55]. This precludes mapping modifications in long native RNA molecules, as can be achieved with nanopore. MS-based RNA sequencing and nanopore sequencing are thus complementary tools for RNome analysis.

### Quality control: ensuring the accuracy of modification-aware sequencing and analysis

The accuracy of epitranscriptomic analysis is determined by the combined impact of errors introduced during experimental procedures and data processing. To ensure high-quality data, experimental design must include an adequate number of replicates, sufficient sequencing depth, and the incorporation of both positive and negative controls. Method-specific data analysis should employ robust statistical frameworks to evaluate the significance of signals at specific sites, accounting for sample size, signal strength, and their relevance within the broader context of all samples, including replicates and controls. Given the diversity of current modification mapping methods, it is challenging to recommend a universal set of parameters for experimental design and data analysis. Therefore, we outline guidelines based on general principles in the following sections.

### Conventional sequencing errors and “molecular input” errors

Base-calling accuracy in Illumina sequencing data typically has an error rate of 0.1–0.5% per nucleotide residue, while nanopore sequencing has only recently reduced its error rates to the single-digit range. While these error rates are not typically a major concern for conventional RNA sequencing, they become critical when using molecular input methods that depend on errors for mapping modifications, as these methods can introduce artifact-based errors, such as false positives and false negatives. To ensure data validity and reliability, it is essential to include a sufficient number of both biological and technical replicates, as well as adequate sequencing depth to optimize the signal-to-noise ratio. The significance of a detected signal is further strengthened by comparisons with positive and negative controls, ideally including at least one of each that represents a “gold standard” or ground truth.

### Data interpretation

Quality control (QC) parameters are essential at multiple levels, including raw data (e.g., fastq files used for downstream analysis) and the analytical pipelines used for mapping modified residues. For raw data, QC criteria can often follow established standards for the respective sequencing technology, such as a Q-score > 30 for Illumina sequencing. The thresholds, however, may vary depending on whether short-read or long-read sequencing technologies are employed. Beyond this, a second layer of QC is needed to evaluate the performance of molecular input methods, which introduce their own characteristic errors. A third layer of QC pertains to computational analysis, assessing the reliability of data interpretation across different epitranscriptomics mapping protocols and pipelines. In some cases, it may be valuable to integrate these QC layers into aggregated error rates or composite metrics that encompass both molecular and computational aspects.

To advance the field, it is imperative to establish a universally accepted set of QC parameters for benchmarking methods. Equally important is the determination of standardized threshold values for these parameters, which could become mandatory for the Human RNome Project. The diversity of existing technologies, as well as those that will emerge during the project, complicates the establishment of universal QC criteria at the raw data level. However, any method must undergo rigorous validation before being deemed suitable for modification calling.

Validation should involve the creation of models evaluated with metrics such as receiver operating characteristic (ROC) curves, area under the curve (AUC), sensitivity (true positive rate), and specificity (true negative rate). Particular attention must be given to minimizing false positive and false negative rates, as these directly impact the reliability of modification detection. Another critical input parameter for these models is an accurate estimate of the expected number of residues for a given modification, as this will influence thresholds for modification calling. Such an integrative and standardized approach to QC will ensure robust and reliable results across diverse epitranscriptomic applications.

Establishing clear guidelines for reporting QC metrics in publications and data repositories is essential for fostering reproducibility and confidence in results. Comprehensive reporting of raw data quality, molecular input performance, and computational reliability will enable consistent practices across studies. Such transparency not only ensures accountability but also facilitates meta-analyses and comparisons, accelerating progress in the field.

### Vision 2025: strategic steps for the next decade

Advancing modification-aware RNA sequencing on an international scale requires both organizational and technical developments. One of the greatest challenges will be achieving consensus within the field on a mandatory set of QC parameters and, even more challenging, establishing universally applicable threshold values. As highlighted earlier, in addition to maintaining a continuously updated overview of methodologies, the field must identify techniques that either deliver the highest throughput with minimal error rates or enable precise quantification of modification levels at specific RNA sites. With these considerations in mind, we outline the following ongoing and future objectives for the Human RNome Project.

### Future goals

#### Short-term

1. Continue developing NGS, nanopore, and MS technologies to (a) expand the repertoire of modifications for NGS and nanopore by developing and refining chemical derivatization methods; (b) expand training datasets and algorithms for nanopore; and (c) increase the sensitivity, LC resolution, and data processing algorithms for MS-based sequencing.
2. Integrate orthogonal technologies (e.g., combinations of methods providing different molecular inputs or alternate sequencing technologies) to confirm RNA modifications with high confidence on native RNAs.
3. Develop and implement robust quality control (QC) protocols for NGS, nanopore, and MS to (a) minimize artifacts, (b) increase statistical power, (c) increase sequencing depth, and (d) assure inter-laboratory consistency.
4. Lay the groundwork for scaling and throughput: (a) multiplexing MS-based sequencing; (b) automation of sequencing library preparation, sample analysis, data processing, and data mining; and (c) inter-laboratory validation.
5. Create user groups to develop, implement, and cross-validate RNA-seq methods. Begin developing or adapting websites and databases for public access to protocols and RNA-seq datasets. Engage international funding bodies to support research and development.

#### Medium-term

1. Develop automated systems for RNA extraction, size- or sequence-based RNA purification, library preparation, sequencing, data processing, and data analysis.
2. Develop and refine computational methods: (a) algorithms to interpret raw sequencing data, distinguish true modification signals from noise, and quantify modifications; (b) standardize modification-calling pipelines with open datasets; and (c) develop rigorous benchmarks to ensure reproducibility.
3. Expand scale of sequencing efforts: Prioritize high-throughput, automated solutions to handle increasing data demands; integrate methods with lower error rates and reliable quantification into streamlined workflows.
4. Expand RNA-seq analyses using cell and RNA targets identified in section I. Apply improved workflows to diverse cell types and RNA populations to create comprehensive modification maps.

#### Long-term

1. Develop new sequencing technologies: (a) design new nanopore pore systems, (b) develop RNA-customized MS ionization, fragmentation, and detection hardware; (c) innovate platforms capable of directly sequencing full-length RNA molecules with single-base resolution and error rates < 0.1%.
2. Continue developing AI and automation technologies: Design AI-driven base-calling algorithms for real-time error correction and precise modification detection.
3. Expand RNA-seq databases and integrate across databases.
4. Expand application of RNA-seq technologies: (a) cells beyond those initially identified as standards for the Human RNome Project (Section I); (b) tissues from animal models; and (c) human clinical samples.

### Guidelines for data analysis, formatting, and storage

**Fig. 4:**
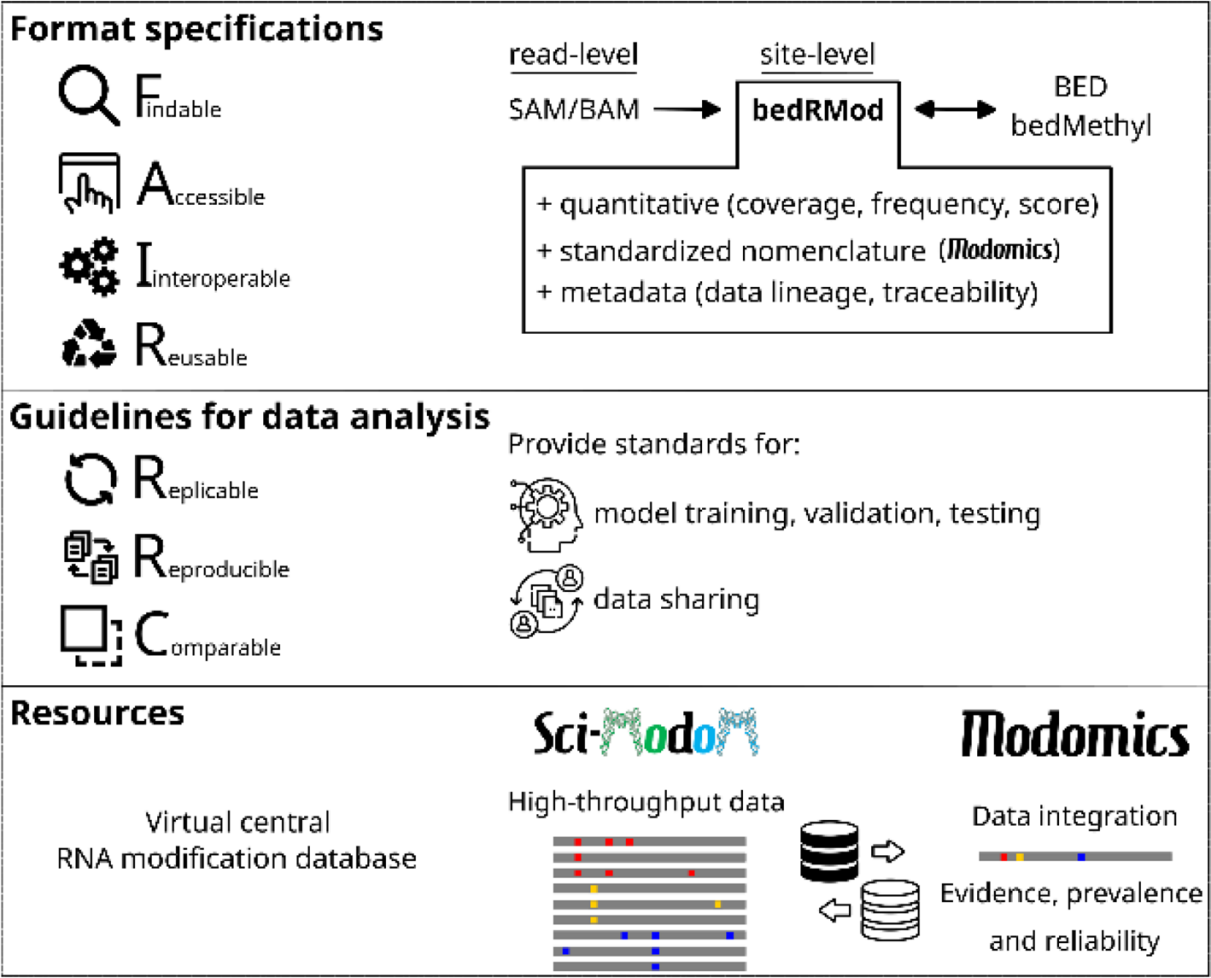
Data handling to ensure long-term and reproducible usage of the data acquired for the Human RNome Project

A tremendous amount of the epitranscriptome sequencing data generated in the last few years has, on the whole, remained unused, because of limited data accessibility, poor findability and reusability. Addressing these gaps could significantly enhance the utility and impact of these data. In this section, we propose FAIR guidelines [59] for data format specifications, model training standards, and protocols for recording and sharing information related to RNA sequences and modifications, applicable to both indirect and nanopore direct RNA sequencing (Fig. 4).

### Data format specifications and nomenclature

The identity, position, and frequency of RNA modifications are derived from large volumes of raw data, typically mapped reads. These analyses depend on method-specific technological expertise, which can vary significantly across approaches. Raw data alone are often not practical when only site-specific modification information is required. Moreover, this information has historically been disseminated through a range of incompatible formats, governed by varying standards, and often accompanied by limited access or incomplete metadata. These challenges have hindered reproducibility and the ability to compare results across studies.

While data formats for raw sequencing data are well-established, no such standardization exists for modification information derived from these data. At a minimum, site-specific RNA modification data should be reported in a straightforward format and include:

1. A standardized naming convention for the modification type.
2. Stoichiometric information, such as the percentage or frequency of the modification.
3. Depth of coverage for the modification site.
4. Quantitative confidence scores indicating the reliability of the modification call.

At the dataset level, metadata should be sufficiently detailed to ensure traceability, reproducibility, and reusability. This requires reliance on standardized nomenclature while maintaining flexibility to include free-text information where necessary. Some of this information has recently been incorporated into the latest SAM/BAM format specifications [60], where nucleotide residue modifications and their quality scores are recorded per-read.

At the per-site level, the recently proposed bedRMod format addresses many of these requirements [32]. This format is analogous to the ENCODE bedMethyl standard [61] and nanopore’s extended bedMethyl format [62] and compatible with the widely used BED (Browser Extensible Data) format. It was developed during the Human Genome Project [60] and approved by the GA4GH Standards Steering Committee and it integrates seamlessly with many command-line tools and genome browsers.

However, a significant barrier to the widespread adoption of the bedRMod format is its dependency on information of nucleotide residue modification from SAM/BAM files, which in turn relies on mapping algorithms. Tools to compile this data into bedRMod format at the site level remain underdeveloped, and current workflows often rely on custom algorithms to extract site-specific information into similar tabulated formats. Addressing this gap with robust, standardized tools will be essential to advancing the use and utility of bedRMod for RNA modification studies.

The RNA modification nomenclature adopted by MODOMICS [12, 13] aligns well with the requirements of the bedRMod format by providing standardized names for RNA modifications. MODOMICS uses a variety of representations, including multi-character alphanumeric codes for single and multiple sequence formats like FASTA, a one-letter Unicode-based code for sequence alignments, and a human-readable alphanumeric code for broader accessibility. Recent updates to MODOMICS have expanded its nomenclature to include synthetic residues, accommodating the growing diversity of RNA modifications in both research and practical applications [12]. This system is instrumental in ensuring compatibility across tools and datasets and holds potential as a foundation for a future standardized nomenclature under IUPAC guidelines.

### Data requirements for training, validation, and testing of RNA modification

Development of RNA modification calling algorithms requires independent datasets for model training, (cross-) validation, and additional datasets for testing and benchmarking established methods. The previous sections have outlined potential biological and synthetic sources, as well as sequencing approaches, for generating these datasets. To ensure relevance, datasets may need to align with the specific focus of interest, whether tRNAs, mRNAs, or rRNAs, as these classes differ significantly in their epitranscriptomic properties and sequence characteristics.

Additionally, datasets should accurately represent the real-world distribution of modified versus unmodified nucleotides the model is expected to encounter. They must approximate the size and complexity of existing transcriptomes and include high-quality annotations for modification classes. For example, modification-free transcripts from in vitro transcription of cDNA derived from six immortalized human cell lines have been used as a robust ground-truth dataset for unmodified mRNA transcriptomes [49]. Similarly, Chan et al. [63] employed random ligation of RNA oligos with known modification statuses to construct longer transcripts with sufficient complexity in both nucleotide composition and modification density, representing another valuable resource for RNA modification research.

### Towards a central RNA modification database

The Human RNome Project seeks to establish guidelines for formatting and sharing RNA sequences and modifications, while also consolidating and integrating the growing volume of high-throughput epitranscriptome data. This effort aims to enhance data accessibility, facilitate the automated discovery of datasets, and optimize data reuse. Sci-ModoM [32] introduces a novel, quantitative framework supported by the bedRMod format, advancing the adoption of FAIR data principles and fostering the use of common standards. These features position Sci-ModoM as a potential cornerstone database for RNA modifications.

Developed in synergy with MODOMICS [12, 13], Sci-ModoM complements this meta-database by offering high-throughput, high-resolution data in a standardized format. Sci-ModoM serves as a centralized platform where modifications from diverse studies can be accessed and compared, while MODOMICS provides a curated repository of RNA sequences enriched with all known modifications, along with detailed metadata on their reliability and prevalence. Together, these resources enable the visualization of modifications within RNA sequences and broaden the utility of epitranscriptome data for research, therapeutic development, and experimental applications. The integration of Sci-ModoM and MODOMICS represents a significant milestone in achieving comprehensive annotation and effective utilization of RNA modifications.

### Future goals

#### Short-term

1. To establish bedRMod as the format for sharing RNA modification data, and to develop the necessary infrastructure and tools to improve interoperability, and to facilitate its use by the community.
2. To establish guidelines and minimal requirements for training, validation, testing, and sharing RNA modification data and software.
3. To make realistic training and validation data available through Sci-ModoM to support the development of new detection methods and algorithms (cf. Future goals of section III).

#### Medium-term

1. To continuously and dynamically annotate novel modifications from the large amount of data available in Sci-ModoM using MODOMICS evidence levels, reliability scores, and prevalence metrics.
2. To enhance the interpretability of transcriptome-wide data accumulated in Sci-ModoM by contextualizing it with broader biochemical, structural, and functional information available in MODOMICS, bridging experimental findings with mechanistic insights.
3. To provide global mirroring and public access, e.g., through collaboration with academic institutions, following the open-access model of Sci-ModoM.

#### Long-term

1. To build on the synergistic development of Sci-ModoM and MODOMICS to establish a virtual central RNA modification database.
2. To establish a standardized data flow allowing users to transition seamlessly from experimental data in Sci-ModoM to comprehensive annotations in MODOMICS.

### The transformative impact of RNA science

The Human RNome Project is a bold and transformative initiative poised to revolutionize diverse sectors, including biomedicine, agriculture, data storage, and global security. By advancing RNA science, this project will deepen our understanding of RNA biology and catalyze groundbreaking innovations, delivering profound societal benefits.

Biomedicine: RNA research has made critical contributions to healthcare, particularly in understanding the biology of RNA viruses like SARS-CoV-2 and other infectious agents. These insights have accelerated the development of RNA-based therapeutics, including mRNA technologies now being adapted for applications such as influenza [64, 65] and malaria prevention [66]. Beyond infectious diseases, RNA-based therapies are transforming treatment paradigms for various conditions. Nusinersen, an antisense oligonucleotide therapy, has significantly improved outcomes for children with spinal muscular atrophy, enabling them to achieve developmental milestones [67, 68]. Inclisiran, an RNA interference-based drug, provides an effective biannual treatment for lowering LDL cholesterol, improving compliance compared to daily regimens [69, 70]. RNA-based therapies continue to advance in oncology, rare diseases, and other fields. The Human RNome Project will support this progress by improving targeting with highly accurate RNA sequences, reducing costs through the production of affordable, high-quality ribonucleotides, including modified forms, bolstering supply chains, and expanding access to RNA therapeutics.

Agriculture: Global food insecurity is a pressing issue affecting millions worldwide. In the USA alone, over 10 million children face hunger, and globally, malnutrition affected 27 million children in 2022. RNA-based technologies offer innovative solutions to address these challenges. Research shows that RNA modifications can enhance crop yields in staples like rice and potatoes [71], improving resilience and productivity. Additionally, RNA interference (RNAi) delivered via high-pressure sprays provides an effective, non-genetically engineered method to combat plant diseases [72, 73]. RNA sequencing technologies will equip plant scientists with powerful tools to improve crop productivity and combat global hunger.

Data storage: RNA presents a transformative approach to ultra-dense, efficient, and scalable data storage, capitalizing on its structural complexity and extensive chemical diversity. Unlike the binary 0,1 system traditionally used for data encoding, RNA’s repertoire of approximately 180 known ribonucleotide modifications vastly expands the encoding alphabet. While the binary system encodes 1 bit of information per symbol, RNA modifications encode approximately 7.49 bits per symbol, enabling a 649% improvement in compression efficiency.

This groundbreaking technology not only addresses the rapidly growing global demand for storage capacity, projected to surpass available resources in the coming decades, but also offers a sustainable and cost-effective alternative. By leveraging RNA’s ability to store dense information in a biochemically compact format, this innovation has the potential to revolutionize data storage while reducing the environmental and financial costs associated with conventional methods.

The Human RNome Project will be at the forefront of this innovation, establishing the necessary infrastructure to produce standard and modified ribonucleotides as foundational components for RNA-based storage. In parallel, the project will drive advancements in sequencing and synthesis technologies to ensure data integrity, reliability, and affordability. These efforts will transition RNA-based storage from theoretical concept to practical reality, opening a new frontier in data technology. By combining unparalleled storage density with innovative compression strategies, RNA-based systems promise to redefine how we store and access the world’s growing digital archives.

Pandemic and biowarfare preparedness: RNA viruses are responsible for nearly half of infectious diseases, including influenza, Ebola, hepatitis A, and COVID-19. Their high mutation rates, up to five times that of DNA viruses, make early detection and control challenging [74, 75]. The Human RNome Project will revolutionize RNA virus sequencing, enabling rapid and accurate identification of emerging pathogens. This capability will enhance global pandemic preparedness, providing the tools needed to respond swiftly to new threats. Moreover, advances in RNA sequencing will be critical for detecting engineered viruses, strengthening defenses against biowarfare, and ensuring global health and security. By enabling rapid, precise detection, the RNome Project will play a vital role in safeguarding against both natural and human-made threats.

In conclusion, the Human RNome Project will drive transformative progress across a range of fields, from health and agriculture to technology and security. By unlocking the full potential of RNA science, this initiative will deepen our understanding of fundamental biological processes and empower innovative solutions to some of humanity’s most pressing challenges. With its far-reaching applications and societal impact, the Human RNome Project promises to be a cornerstone of twenty-first-century innovation, paving the way for a healthier, more resilient future.

## Data Availability

No datasets were generated or analysed during the current study.
